# Supplementary material for: Altered microRNA Transcriptome in Cultured Human Liver Cells upon Infection with Ebola Virus
Source: Int J Mol Sci. 2021 Apr 6;22(7):3792. doi: 10.3390/ijms22073792 (PMC8038836; doi:10.3390/ijms22073792)
Supplement: Supplementary file 1 [file ijms-22-03792-s001.zip › Supplementary_File/ijms-1147524-supp.docx]

Supplementary Data File

**Altered microRNA Transcriptome in Cultured Human Liver Cells upon Infection with Ebola Virus**

**Citation:** Diallo, I.; Ho, J.; Laffont, B.; Laugier, J.; Benmoussa, A.; Lambert, M.; Husseini, Z.; Soule, G.; Kozak, R.; Kobinger, G.P.; et al. Altered
microRNA Transcriptome in
Cultured Human Liver Cells upon Infection with Ebola Virus. *Int. J. Mol. Sci.* **2021**, *22*, x. https://doi.org/10.3390/xxxxx

Academic Editor: Andreas Burkovski

Received: date

Accepted: date

Published: date

**Publisher’s Note:** MDPI stays neutral with regard to jurisdictional claims in published maps and institutional affiliations.

**Copyright:** © 2021 by the authors. Submitted for possible open access publication under the terms and conditions of the Creative Commons Attribution (CC BY) license (http://creativecommons.org/licenses/by/4.0/).

**Idrissa Diallo ^1^, Jeffrey Ho ^1^, Benoit Laffont ^1^, Jonathan Laugier ^1^, Abderrahim Benmoussa ^1^, Marine Lambert ^1^,
Zeinab Husseini ^1^, Geoff Soule ^2^, Robert Kozak ^2,3^, Gary P. Kobinger ^1,4^ and Patrick Provost ^1,5,^***

*^1^*	CHU de Québec Research Center, Department of Microbiology, Infectious Diseases and Immunology,
Faculty of Medicine, Université Laval, Quebec, QC, Canada; Idrissa.Diallo@crchudequebec.ulaval.ca (I.D.); jeffrey.ho@crchudequebec.ulaval.ca (J.H.); laffont.benoit@gmail.com (B.L.); laugierjonathan@yahoo.fr (J.L.); abderrahim.benmoussa@umontreal.ca (A.B.); Marine.Lambert@crchudequebec.ulaval.ca (M.L.); zeinab.husseini@crchudequebec.ulaval.ca (Z.H.); Gary.Kobinger@crchudequebec.ulaval.ca (G.P.K.)

^2^	Special Pathogens Program, National Microbiology Laboratory, Public Health Agency of Canada; geoff.soule@phac-aspc.gc.ca (G.S.); rob.kozak@sunnybrook.ca (R.K.);

^3^	Department of Laboratory Medicine & Molecular Diagnostics, Division of Microbiology, Sunnybrook Health Sciences Centre, Toronto, Ontario, Canada

^4^	Special Pathogens Program, Department of Medical Microbiology, National Microbiology Laboratory,
Public Health Agency of Canada, Department of Immunology, University of Manitoba, Winnipeg

^5^	CHUQ Research Center/CHUL Pavilion, 2705 Blvd Laurier, Room T1-65, Quebec, QC G1V 4G2, Canada

*	Correspondence: patrick.provost@crchudequebec.ulaval.ca; Tel.: 1-418-525-4444 (ext. 48842)

**Figure S1. Adapter-trimmed reads length distribution in Huh7 cells infected or not with Ebola virus.** The total adaptor-trimmed read count for each length (16-30 nt) was measured for uninfected (black), Mayinga (blue), Makona (gold), and Reston (gray) at 24 h and 96 h post-infection.

**Table S1. The read counts at the data processing stages.** The total numbers of the reads (cleaned after 3’ adapter trimming and passed Solexa CHASTITY quality filter) at the sequencing data processing stages are listed for each sample. Reads aligned to known human pre-miRNAs were expressed in counts and as percentage relative to the adapter-trimmed reads. The differential is the subtraction of the reads at the late stage (96 h) and the initial stage (24 h).

**Figure S2. Venn diagrams showing unique and common miRNAs detected (by RNA-Seq) in Huh7 cells infected with 3 ebolavirus strains (Mayinga, Makona, Reston).**

**Figure S3. Bar plot showing the top 10 fold enrichment value of the significant enrichment GO terms at 24 h post-infection.** Filtered targets of upregulated miRNAs at early stage (24 h) of infection with Mayinga, Makona and Reston subjected to GO functional analysis (Biological Process).

**Figure S4. Bar plot showing the top 10 Fold Enrichment value of the significant enrichment terms at 24 h post-infection**. Filtered targets of upregulated miRNAs at early stage (24h) of infection with Mayinga, Makona and Reston variants subjected to GO functional analysis (molecular function).

**Figure S5. Bar plot showing the top 10 significantly enriched KEGG pathways of genes targeted by upregulated miRNAs at 24 h and 96 h post-EBOV infection.**

**Figure S6. Bar plot showing the top 10 Fold Enrichment value of the significant enrichment terms at 96 h post-infection.** Filtered targets of upregulated miRNAs at late stage (96 h) of infection with Mayinga, Makona and Reston subjected to GO functional analysis (biological process).

**Figure S7. Bar plot showing the top 10 Fold Enrichment value of the significant enrichment terms at 96 h.** Filtered targets of upregulated miRNAs at late stage (96 h) of infection with Mayinga, Makona and Reston variants subjected to GO functional analysis (molecular function).

**Figure S8. Bar plot showing the top 10 Fold Enrichment value of the significant enrichment terms at 24 h post-infection.** Filtered targets of downregulated miRNAs at early stage (24 h) of infection with Mayinga, Makona and Reston subjected to GO functional analysis (biological process).

**Figure S9. Bar plot showing the top 10 Fold Enrichment value of the significant enrichment terms at 24h post-infection.** Filtered targets of downregulated miRNAs targets at early stage (24 h) of infection with Mayinga, Makona and Reston variant subjected to GO functional analysis (Molecular function).

**Figure S10. Bar plot showing the top 10 significantly enriched KEGG pathways of genes targeted by downregulated miRNAs at 24 h and 96 h post-EBOV infection.**

**Figure S11. Bar plot showing the top 10 Fold Enrichment value of the significant enrichment terms at 96 h post-infection.** Filtered targets of downregulated miRNAs at late stage (96 h) of infection with Mayinga, Makona and Reston subjected to GO functional analysis (biological process).

**Figure S12. Bar plot showing the top 10 Fold Enrichment value of the significant enrichment terms at 96h post-infection.** Filtered targets of downregulated miRNAs at late stage (96 h) of infection with Mayinga, Makona and Reston variant subjected to GO functional analysis (molecular function).

**Figure S13. Downregulated miRNAs which can potentially interact with ZEBOV or RESTV.** The sequence of the top downregulated host miRNA and those naturally abundant found were aligned with ZEBOV and RESTON genome using RNA22. Alignments with the lowest free energy in Kcal/mol and the highest probability (p≤0.05) were reported along with the leftmost position in nts of the alignment on the genome.

**24H**

A. **MAYINGA**

**96H**

B. **MAKONA**

**24H**

**96H**

C. **RESTON**

**24H**

**96H**

**Figure S14.** **Upregulated miRNA-target gene network constructed with miRNet**. Upregulated DE-miRNAs following A) Mayinga, B) Makona or C) Reston infection were subjected to the miRNet web-based tool to obtain their downstream target genes. The yellow and pink nodes correspond respectively to genes involved in KEGG pathways and to regular genes targeted by miRNAs (which are represented by the blue squares). The functional annotation table is resumed in the supplementary file F.

**24H**

A. **MAYINGA**

**96H**

B. **MAKONA**

**24H**

**96H**

C. **RESTON**

**24H**

**96H**

**Figure S15.** **Downregulated miRNA-target gene network constructed with miRNet**. Downregulated DE-miRNAs following A) Mayinga, B) Makona or C) Reston infection were subjected to the miRNet web-based tool to obtain their downstream target genes. The yellow and pink nodes correspond respectively to genes involved in KEGG pathways and to regular genes targeted by miRNAs (which are represented by the blue squares).The functional annotation table is resumed in the supplementary file F.

Materials and Methods

**Experiment workflow**

RNA Sample QC The purity and concentration of total RNA samples were determined with NanoDrop ND-1000. Results were provided in Sample QC report.

Sequencing Library Preparation Total RNA of each sample was sequentially ligated to 3’ and 5’ small RNA adapters. cDNA was then synthesized and amplified using Illumina’s proprietary RT primers and amplification primers. Subsequently, ~130 -150 bp PCR amplified fragments were extracted and purified from the PAGE gel. And finally, the completed libraries were quantified by Agilent 2100 Bioanalyzer.

Cluster Generation The samples were diluted to a final concentration of 8 pM and cluster generation was performed on the Illumina cBot using TruSeq Rapid SR cluster kit (#GD-402-4001, Illumina), following manufacturer’s instructions.

Sequencing on Illumina NextSeq 500 Sequencing was performed on Illumina NextSeq 500 using TruSeq Rapid SBS Kits (#FC-402-4002, Illumina), according to the manufacturer’s instructions

Data Collection and Analysis

**Flowchart of Data Analysis**

Clean reads: Illumina HiSeq Real-time base calling and quality filtering.

Adaptor trimmed reads: Removal of adaptor sequence from the reads.

Alignment to reference genome: Classification and distribution of small RNAs. Annotation and tag counts of miRNAs.

Normalization: Tags per million of aligned miRNA (TPM)

Standard analysis: Differentially expressed miRNAs and novel miRNA prediction

| mRNA targets | Sequences (5’-3’) | A. T. (*C) | Accessions |
| --- | --- | --- | --- |
| ACTB | FW: GATTCCTATGTGGGCGACGA  RV: AGGTCTCAAACATGATCTGGGT | 55 | NM_001101.5 |
| GP ZEBOV | FW: TTTTCAATCCTCAACCGTAAGGC  RV: CAGTCCGGTCCCAGAATGTG | 50 | NC_002549.1 |
| GP RESTV | FW: TCCAAGGCTCGAACAACTCC  RV: CGCTTTTGTTTCCTGGTGGG | 52 | NC_004161.1 |
| IL-6 | FW: ATCATCACTGGTCTTTTGGAGTTT  RV: GCATCTAGATTCTTTGCCTTTTTC | 55 | NM_000600.5 |
| IL-8 | FW: CTGATTTCTGCAGCTCTGTGT  RV: GCTCTCTTCCATCAGAAAGCT | 55 | NM_000584.4 |
| IL-1b | FW: AGTGAAATGATGGCTTATTACAGTG  RV: GCAGTTCAGTGATCGTACAGGT | 58 | NM_000576.3 |
| TNF | FW: CCCCAGGGACCTCTCTCTAA  RV: CTCAGCTTGAGGGTTTGCTAC | 60 | NM_000594.4 |
| ICAM1 | FW: AGCTTCGTGTCCTGTATGGC  RV: TTTTCTGGCCACGTCCAGTT | 54 | NM_000201.3 |
| VCAM1 | FW: TGGATAATGTTTGCAGCTTCTCA  RV: TTCGTCACCTTCCCATTCAGT | 52 | NM_001078.4 |
| F2 | FW: CCGAAGGCTCCAGTGTGAAT  RV: AGCTGCACAGCTGAGTTGAA | 53 | NM_000506.5 |
| F3 | FW: ACACCTTACCTGGAGACAAACC  RV: GGTTAACTGTTCGGGAGGGA | 54 | NM_001993.5 |
| F8 | FW: CAAAGTGATCTCGGTGAGCTG  RV: GTAGGACCTAGCAGACCCATC | 51 | NM_000132.4 |
| F10 | FW: TCAAGGTGAGGGTAGGGGAC  RV: GGAAGGTGATGGGGGTCTTG | 53 | NM_000504.4 |
| Casp-3 | FW: GCAGCAAACCTCAGGGAA  RV: TGTCGGCATACTGTTTCAGCA | 58 | NM_001354777.2 |
| Bax | FW: TGGCAGCTGACATGTTTTCTGA  RV: CAGGGACATCAGTCGCTTCAGT | 58 | NM_001291428.2 |
| Bcl-2 | FW: TTTGAGTTCGGTGGGGTCAT  RV: TGACTTCACTTGTGGCCCAG | 58 | NM_000633.3 |

**Table S2: Primer sequences used in RT-qPCR assays in Huh7 cells. A. T= Annealing temperature (Celsius)**
